# 18^th^ International Summer School of Neurology in conjunction with the 13^th^ European Teaching Course on Neurorehabilitation

**DOI:** 10.25122/jml-2023-1031

**Published:** 2023-11

**Authors:** Stefana-Andrada Dobran, Alexandra Gherman, Dafin Mureşanu

**Affiliations:** 1 RoNeuro Institute for Neurological Research and Diagnostic, Cluj-Napoca, Romania; 2 Department of Neuroscience, Iuliu Hatieganu University of Medicine and Pharmacy, Cluj-Napoca, Romania

## INTRODUCTION

**Figure 1:**
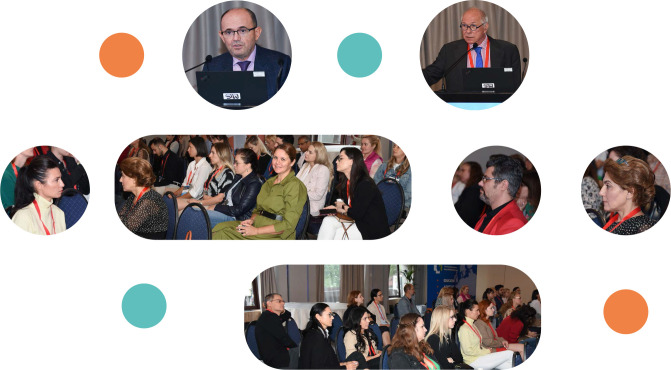
First row: Prof. Dafin Muresanu, EFNR President; Prof. Volker Hömberg, WNFR President; second and third rows: photos of the speakers and participants.

The **18^th^ International Summer School of Neurology, held in conjunction with the 13^th^ European Teaching Course on Neurorehabilitation** took place between 15-16 September, 2023. The events were hosted in a hybrid format in Poiana Brasov, Brasov, Romania, bringing together over 150 online and on-site participants and well-known speakers (Figure 1) who tackled some of the most pressing topics in neurology and neurorehabilitation.

The scientific pursuits were organized by the Society for the Study of Neuroprotection and Neuroplasticity (SSNN) and coordinated by Prof. Dafin F. Muresanu, President of the European Federation of Neurorehabilitation Societies (EFNR) President, Prof. Volker Hömberg, President of the World Federation for Neurorehabilitation (WFNR), and Prof. Natan M. Bornstein, Past Vice-President of the World Stroke Organization (WSO) and Chairman of the Israeli Stroke Society. The events provided a platform for the dissemination of insights and knowledge on neurorehabilitation, neurotraumatology, migraines, Parkinson's disease (PD), multiple sclerosis, stroke, vertigo, dementia, and myasthenia gravis.

An array of national and international speakers showcased compelling presentations regarding pivotal themes within neurology, to ultimately enhance educational opportunities and refine the pathways for clinical practice in this field. These included Dr. Daniela Anghel (Romania), Prof. Rodica Bălașa (Romania), Dr. Dana Boering (Germany), Dr. Adriana Dulămea (Romania), Dr. Amalia Ene (Romania), Prof. Volker Hömberg (Germany), Prof. Tudor Lupescu (Romania), Dr. Ayghiul Mujdaba-Elmi (Romania), Prof. Dafin F. Mureșanu (Romania), Prof. Horia Nicolae (Romania), Prof. Cristian Falup-Pecurariu (Romania), Prof. Caterina Pistarini (Italy), Prof. Bogdan O. Popescu (Romania), Dr. Adina Roceanu (Romania), Dr. Adina Stan (Romania), Dr. Ioana Stănescu (Romania), Dr. József Szász (Romania), Dr. Elena Terecoasă (Romania), Prof. Cristina Tiu (Romania). In essence, both events addressed critical subjects and fostered advancements in the understanding and application of neurology. By focusing on pressing topics, the two developments sought to empower healthcare professionals with the latest knowledge and tools to improve their clinical practice.

On the first day, Professors Volker Hömberg (Germany) and Dafin F. Mureșanu (Romania) delivered a warm welcome address to kickstart the proceedings. Following this, they presided over the first session, which showcased discussions on brain reserve, pharmacological options post-brain injury, assessment tools for functionality, disability, and rehabilitation, as well as the concept of motivation within multidisciplinary approaches. The agenda continued with a keynote lecture by Prof. Volker Hömberg, focusing on critical elements for future developments in the field of neurorehabilitation. Subsequently, the second session, chaired by Dr. Dana Boering (Germany) and Prof. Rodica Bălașa (Romania), delved into the intricacies of migraines. This session addressed the current classification and diagnosis of migraines, eligibility criteria for patients suitable for anti-calcitonin gene-related peptide monoclonal antibodies, the pathology of migraines, and the mechanisms of action of anti-CGRP treatments. The third session brought into the spotlight migraine treatment options. In contrast, the fourth session, chaired by Prof. Cristian Falup-Pecurariu and Dr. Ioana Stănescu from Romania, presented in-depth explorations of PD, multiple sclerosis, vertigo, and ischemic stroke.

On the second day, Prof. Dafin F. Mureșanu and Prof. Bogdan O. Popescu lectured on guidelines and recommendations for infusion therapies for PD and strategies related to the continuous dopaminergic stimulation concept. Moving on, Dr. Amalia Ene addressed the optimization of ON time throughout the progression of PD, while Dr. József Szász discussed the management of advanced PD. Following this session, participants engaged in a practical workshop that focused on the hands-on aspects of subcutaneous continuous apomorphine therapy.

The sixth session, chaired by Dr. Amalia Ene and Dr. József Szász from Romania, highlighted therapeutic approaches for dystonias, the evaluation and treatment of post-stroke spasticity, and the intricate challenges associated with diagnosing acute dementia. Concluding the day, the events featured three captivating presentations on fatigue, the treatment of uncontrolled PD, and clinical approaches and therapies in myasthenia gravis, offering attendees a deeper understanding of common neurological affections.

These academic events were organized by a consortium of institutions and entities including the RoNeuro Institute for Neurological Research and Diagnostic, the Foundation of the Journal for Medicine and Life, the Foundation of the Society for the Study of Neuroprotection and Neuroplasticity, Tel Aviv University, “Iuliu Haţieganu” University of Medicine and Pharmacy Cluj-Napoca, the Romanian Academy, the Romanian Academy of Medical Sciences, and the Academy for Multidisciplinary Neurotraumatology (AMN). It proudly partnered with international organizations such as the European Federation of Neurological Societies (ENFR), the World Federation for NeuroRehabilitation (WFNR), the Foundation for the Study of Nanoneurosciences and Neuroregeneration, and the Romanian Society of Neurology. Additionally, the proceedings received support from industry leaders, such as Teva, AstraZeneca, Viatris, Stada, Ipsen, and Merck.

**Figure 2:**
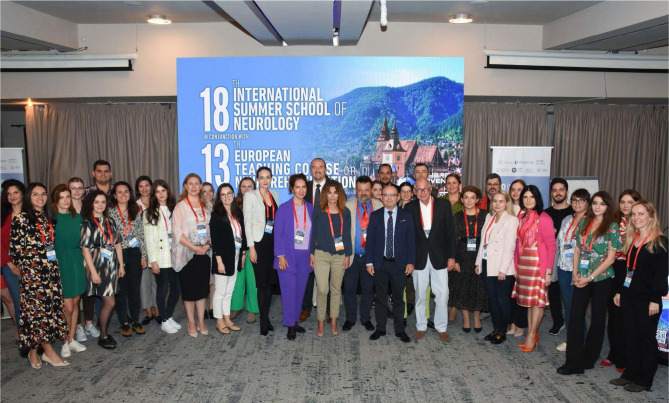
Participants and speakers from the 18^th^ International Summer School of Neurology in conjunction with the 13^th^ European Teaching Course on Neurorehabilitation

The **18^th^ International Summer School of Neurology in conjunction with the 13^th^ European Teaching Course on Neurorehabilitation** (Figure 2) highlighted the importance of multidisciplinary collaboration in advancing science and research. After several restraining years due to the COVID-19 pandemic, the events offered a well-needed platform to forge connections and engage with a dynamic audience, aimed at improving the state of neurology, neurorehabilitation, and associated sciences.

As such, we hope that all potential partners, both academic and from the field industry, would show the same or even higher interest in participating in our future academic events and contribute to developing further new scientific avenues.

